# Inflammatory Pseudotumor of the Skull Base Mimicking Neoplasm: A Rare Case of Trigeminal Neuropathy

**DOI:** 10.7759/cureus.90297

**Published:** 2025-08-17

**Authors:** Niveditha Apparasu, Anudeep Surendranath, Anusha Reddy Muddasani, Abhishek Kengen, Bharath Kumar Jakka

**Affiliations:** 1 Neurology, Kamineni Academy of Medical Sciences and Research Center, Hyderabad, IND; 2 Neurology, CHI St. Vincent Hot Springs, Hot Springs, USA; 3 Internal Medicine, University of Arkansas, Arkansas, USA; 4 Neurology, CHI Memorial Hospital, Chattanooga, USA; 5 Internal Medicine, Baptist Medical Center South, Montgomery, USA

**Keywords:** anaplastic lymphoma kinase, immunoglobulin g4, inflammatory myofibroblastic tumor, inflammatory pseudotumor, trigeminal neuropathy

## Abstract

Trigeminal neuropathy is a rare presenting symptom of inflammatory pseudotumor (IPT), a benign yet locally aggressive lesion with significant diagnostic challenges. A 55-year-old woman presented with right-sided facial pain and numbness. Magnetic resonance imaging (MRI) revealed a T2-hypointense, enhancing lesion in the right parasellar region involving Meckel’s cave and the trigeminal ganglion, mimicking schwannoma or meningioma. Surgical resection and histopathology confirmed IPT, characterized by polyclonal lymphoplasmacytic infiltration and absence of neoplastic or infectious etiology. Central nervous system (CNS) involvement of IPT is rare, and its radiologic mimicry of neoplasms complicates diagnosis, with definitive identification requiring histopathologic and immunohistochemical analysis. In this case, anaplastic lymphoma kinase (ALK) and immunoglobulin G4 (IgG4) negativity ruled out inflammatory myofibroblastic tumor (IMT) and IgG4-related disease. The patient responded well to corticosteroid therapy. IPT should be considered in the differential diagnosis of skull base lesions, and a combination of surgical and corticosteroid therapy can achieve symptom resolution and minimize recurrence.

## Introduction

Inflammatory pseudotumors (IPTs) are etiologically enigmatic, nosologically confusing, and biologically often unpredictable. They are characterized histologically by the presence of acute and chronic inflammatory cells with a variable fibrous response [1]. In the head and neck, they most commonly occur in the orbit or, more rarely, at the skull base [2]. An IPT is a rare, non-neoplastic, idiopathic lesion first described by Brunn in 1939 for its ability to clinically and radiographically mimic malignant neoplasms [3]. It is often considered a tumefactive lesion with prominent fibroblastic proliferation and an inflammatory component, behaving in a locally benign or aggressive manner while clinically and radiologically resembling a neoplastic process. The broad diagnostic category of IPT encompasses entities ranging from reactive inflammatory conditions to true neoplasms. Importantly, it must be differentiated from related conditions such as inflammatory myofibroblastic tumor (IMT) and IgG4-related sclerosing disease [4]. Histopathologically, IPTs consist of spindle-shaped fibroblasts, dense collagen, and a polymorphic infiltrate of lymphocytes and plasma cells.

## Case presentation

A 55-year-old female with a past medical history of hypertension, diabetes mellitus, dyslipidemia, and hypothyroidism, but no history of migraine headache, presented with a one-week history of right periorbital and right frontal headache and right retro-orbital pain. She also had associated numbness in the gum area of the right upper alveolar margin, right angle of the mouth, and upper lip on the right side. She had no associated weakness or numbness in any of her limbs. She had no problems with speech or vision. The headache progressively worsened and was associated with vomiting. She went to the emergency room. A computed tomography (CT) scan of the head without contrast was normal. She was sent home with morphine.

On examination in the office, speech fluency, articulation, and comprehension were normal. Pupils were small but reactive to light; fundi were normal, and there was no gaze paresis. The corneal reflex was intact. Bilateral tactile sensation was intact for the upper and lower face despite subjective numbness. The remainder of the neurological examination, including cranial nerves, peripheral strength, tone, deep tendon reflexes, and plantar reflex, was normal.

**Figure 1:**
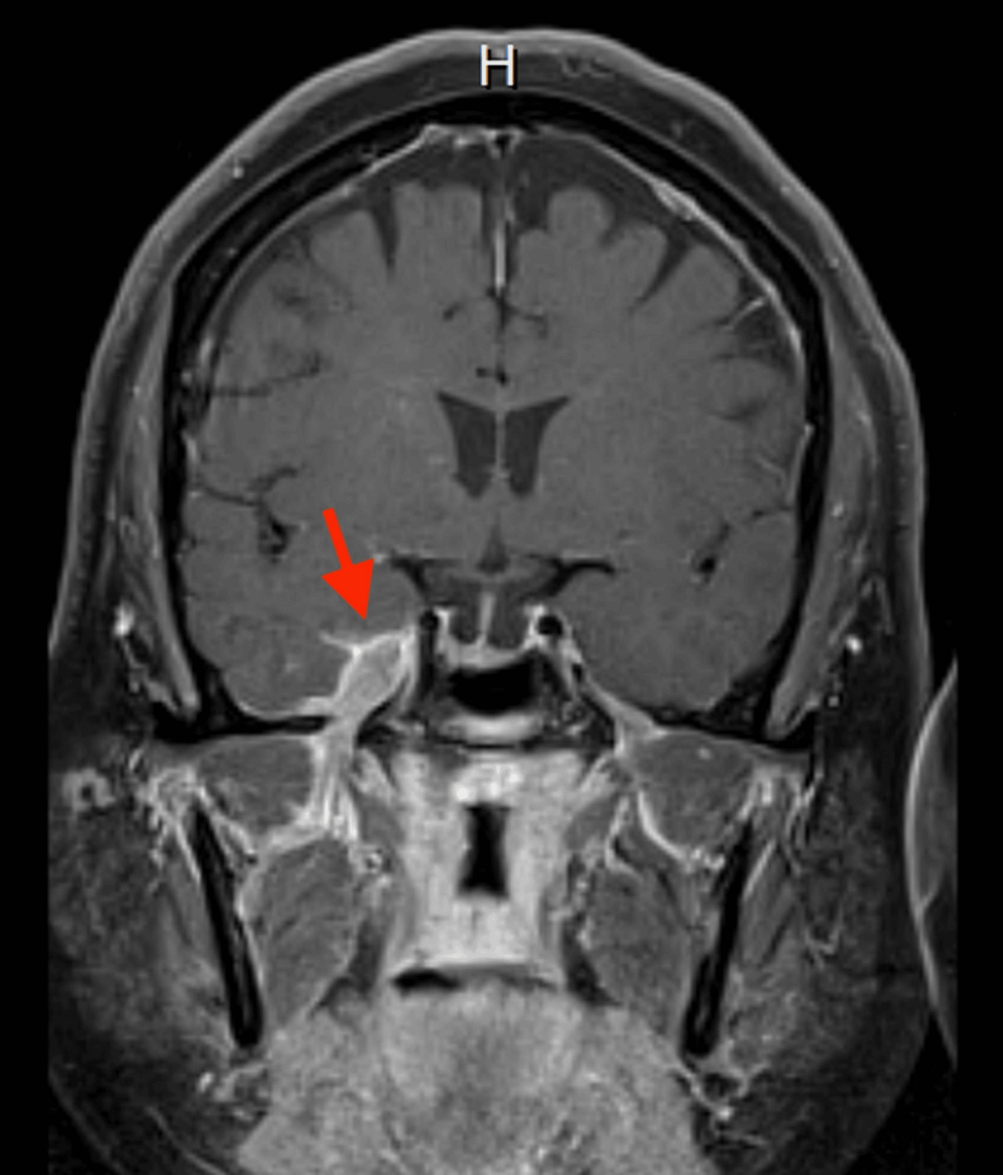
Contrast-enhanced MRI revealing enhancing lesion in the right parasellar region involving Meckel's cave.

An MRI with contrast showed a contrast-enhancing lesion in the right parasellar region involving the trigeminal ganglion. It measured approximately 3.4 cm anteroposteriorly, 1.7 cm cranio-caudally, and 1.7 cm transversely (Figure 1).

It was reported as potentially neoplastic, with differential diagnoses including schwannoma of the trigeminal nerve or a sphenoid wing meningioma.

The patient underwent a right temporal craniotomy for mass removal. Pathologic analysis showed cerebrum (S100 and glial fibrillary acidic protein (GFAP) positive) and chronic inflammatory cell infiltrate composed of polyclonal B and T cells, confirmed with B-cell and T-cell receptor gene rearrangement analysis. A few plasma cells, eosinophils, and occasional neutrophils were also present. There was no evidence of neoplasia or granulomatous disease. Special stains for acid-fast bacilli (Ziehl-Neelsen) and fungi (Grocott Methenamine Silver (GMS) stain) were negative.

The patient was started on dexamethasone (Decadron) after surgery and had significant relief of facial pain. She continued to have mild, subjective numbness along the superior alveolar margin and the right upper lip.

## Discussion

The pathogenesis of IPT remains controversial. IPT most commonly affects the lungs and orbits but can occur in virtually any anatomical location and across all age groups [5,6]. Although initially considered reactive, recent findings of clonal cytogenetic abnormalities and anaplastic lymphoma kinase (ALK) expression in inflammatory myofibroblastic tumors (IMTs) suggest a neoplastic origin for some lesions [6]. The term IMT has therefore been proposed as a neoplastic subset of IPTs, distinguished by features such as ALK gene rearrangement and p53 expression [7,8]. Conversely, lesions such as lymph node and splenic IPTs are considered non-neoplastic and biologically distinct from IMTs, despite their overlapping histologic features [7]. IPTs may also overlap with idiopathic fibro-sclerosing disorders, including retroperitoneal fibrosis, sclerosing mesenteritis, and immunoglobulin G4 (IgG4)-related sclerosing disease [4].

Clinical presentation is variable and may include fever, weight loss, and localized mass effects [6,7]. Central nervous system (CNS) involvement is exceedingly rare, particularly in the absence of systemic disease. Intracranial IPTs often appear as dural-based or parenchymal masses and may occasionally cause cranial neuropathies, especially involving the lower cranial nerves (IX, X, XI, and XII) [9]. Skull base and intracranial IPTs involving cranial nerves - such as the trigeminal nerve - are particularly uncommon, comprising fewer than 15% of all non-orbital head and neck IPTs [1,3,7].

Radiologically, IPTs often mimic malignancy due to features such as T2 hypointensity, homogeneous contrast enhancement, and mass effect, making preoperative diagnosis challenging. However, some imaging characteristics - including T2 hypointensity, dural enhancement, and the absence of diffusion restriction - may favor an inflammatory over a neoplastic etiology.

MRI plays a pivotal role in narrowing the differential diagnosis of skull base lesions. IPT classically demonstrates iso- to hypointense signal on T1-weighted images and low-to-isointense signal on T2-weighted images, attributed to its high cellularity and fibrous stroma. Post-contrast sequences typically reveal avid, homogeneous enhancement. A distinguishing feature is its poorly circumscribed, infiltrative margins with extension along fascial planes and into adjacent soft tissues, without evidence of frank necrosis. IPT may also demonstrate associated dural thickening (pachymeningitis) but lacks the aggressive bone erosion seen in malignant lesions, showing instead smooth remodeling or no osseous change.

In comparison, meningiomas often present as well-circumscribed, extra-axial masses with a broad-based dural attachment and the classic “dural tail” sign on contrast-enhanced MRI. They are frequently hyperintense on T2-weighted images, and adjacent hyperostosis is a common feature, reflecting slow tumor growth.

Schwannomas, particularly trigeminal schwannomas, tend to cause expansion of neural foramina and remodeling of adjacent bone rather than infiltration. They often display a heterogeneously hyperintense signal on T2-weighted images due to cystic degeneration and demonstrate heterogeneous enhancement after contrast administration. The “target sign” (central low T2 signal with peripheral high signal) may be seen in larger lesions.

Lymphomas in the skull base region typically appear iso- to hypointense on T1 and iso- to hypointense on T2, but, unlike IPT, they exhibit marked restricted diffusion on diffusion-weighted imaging (DWI) due to their high nuclear-to-cytoplasmic ratio. They enhance homogeneously but usually present with sharply demarcated margins and less soft tissue infiltration compared to IPT.

Metastatic lesions often appear as multiple foci with variable signal characteristics depending on the primary tumor type. They frequently show heterogeneous enhancement, more aggressive bone destruction, and surrounding edema, which is less typical for IPT.

Granulomatous diseases, such as tuberculosis or sarcoidosis, can mimic IPT both clinically and radiologically. However, they often present with more prominent leptomeningeal enhancement and may be associated with systemic findings detectable on whole-body imaging.

In the presented case, the lesion’s T2 hypointensity, homogeneous post-contrast enhancement, ill-defined infiltrative margins, and absence of significant bone destruction were more in keeping with IPT than with the neoplastic entities in the differential diagnosis. These radiological distinctions, when integrated with the clinical presentation and histopathology, allow for a more confident preoperative diagnosis.

Fluorodeoxyglucose positron emission tomography (FDG-PET) imaging has demonstrated potential for assessing disease activity and treatment response [3], although it was not utilized in our case. MRI remains the preferred imaging modality, particularly for detecting subtle nerve involvement and evaluating soft tissue detail. Advanced imaging, such as magnetic resonance spectroscopy and perfusion MRI, may aid in distinguishing IPT from neoplastic lesions by demonstrating hypoxia, lactate accumulation, and low cerebral blood volume [10].

Histopathologic confirmation remains the gold standard for diagnosis. In our case, polyclonal B and T cells with scattered eosinophils and the absence of neoplastic markers supported a diagnosis of IPT. The lesion was negative for ALK, IgG4, and other tumor-specific markers, ruling out IMT and IgG4-related disease [3,11,12]. The inflammatory infiltrate was CD20 and CD3-positive, confirming a reactive immune response.

Etiologically, IPT has been linked to infectious agents (e.g., Epstein-Barr virus (EBV) [13,14], actinomyces [15], human immunodeficiency virus (HIV)), autoimmune disorders (e.g., Sjögren’s syndrome, neuro-Behçet’s disease), and cytokine-mediated immune dysregulation [13,14]. A significant number of IPTs exhibit elevated immunoglobulin levels or serologic markers of immune activation. EBV has been identified in up to 40% of IPT cases [13,14], supporting a viral hypothesis in some instances.

Management of IPT depends on site, severity, and histopathologic findings. For orbital IPTs, corticosteroids are often sufficient. In contrast, skull base and CNS IPTs typically require surgical resection for both diagnostic and therapeutic purposes. Postoperative corticosteroids improve outcomes and reduce inflammation. Maintenance therapy for six months or longer may be necessary to prevent recurrence [16]. Intralesional steroids, radiotherapy, and targeted therapies, including rituximab and crizotinib, are viable options in steroid-refractory or recurrent cases.

Differential diagnoses include granulomatous diseases (e.g., sarcoidosis, Wegener’s granulomatosis), neoplasms (e.g., schwannoma, lymphoma, meningioma), and infectious etiologies. All were excluded in our case through comprehensive imaging, microbiologic, and immunohistochemical analyses (Table 1). FDG-PET imaging, although not used in our case, may help monitor disease activity and therapeutic response.

**Table 1:** Histopathologic and immunohistochemical findings supporting the diagnosis of inflammatory pseudotumor. ALK = anaplastic lymphoma kinase; IgG4 = immunoglobulin G4; CD = cluster of differentiation; IMT = inflammatory myofibroblastic tumor; IPT = inflammatory pseudotumor

Parameter	Finding
Histopathology	Polyclonal B and T cells with scattered eosinophils; absence of neoplastic markers
ALK (anaplastic lymphoma kinase)	Negative
IgG4 (immunoglobulin G4)	Not significantly elevated
Other tumor-specific markers	Negative, ruling out inflammatory myofibroblastic tumor (IMT) and IgG4-related disease
Inflammatory infiltrate markers	CD20 positive (B cells); CD3 positive (T cells)
Interpretation	Findings support inflammatory pseudotumor (IPT) diagnosis; consistent with reactive immune response and exclusion of malignancy

This case highlights the importance of considering IPT in skull base lesions mimicking neoplasms, particularly when imaging shows T2 hypointensity, dural thickening, and cranial nerve involvement. Timely diagnosis and combined surgical-corticosteroid treatment can provide durable symptom relief.

## Conclusions

This case emphasizes the importance of considering IPT in the differential diagnosis of skull base lesions, particularly in patients presenting with trigeminal neuropathy and radiologic features suggestive of neoplasm. Definitive diagnosis requires histopathologic and immunohistochemical evaluation to exclude neoplastic, infectious, and immune-mediated entities. Early multidisciplinary involvement, prompt surgical intervention when indicated, and timely initiation of corticosteroid therapy may improve outcomes and help avoid unnecessary radical resections.
